# The effects of weight loss interventions on children and adolescents with non‐alcoholic fatty liver disease: A systematic review and meta‐analysis

**DOI:** 10.1002/osp4.758

**Published:** 2024-04-26

**Authors:** Mohammad Hassan Sohouli, Seyedeh Elaheh Bagheri, Somaye Fatahi, Pejman Rohani

**Affiliations:** ^1^ Student Research Committee Department of Clinical Nutrition and Dietetics Faculty of Nutrition and Food Technology Shahid Beheshti University of Medical Sciences Tehran Iran; ^2^ Pediatric Gastroenterology and Hepatology Research Center Pediatrics Centre of Excellence Children's Medical Center Tehran University of Medical Sciences Tehran Iran; ^3^ Tehran University of Medical Sciences Tehran Iran; ^4^ Pediatric Gastroenterology, Hepatology, and Nutrition Research Center Research Institute for Children's Health Shahid Beheshti University of Medical Sciences Tehran Iran

**Keywords:** adolescent, anthropometry, children, glycemic index, lipid profile, liver profile, non‐alcoholic fatty liver disease, weight loss

## Abstract

**Background:**

Overall, there is conflicting evidence regarding the beneficial effects of optimal lifestyle modification, particularly weight loss interventions, with nonalcoholic fatty liver disease (non‐alcoholic fatty liver disease (NAFLD)). Therefore, this study investigated the effects of weight loss interventions on laboratory and clinical parameters in children and adolescents with NAFLD.

**Methods:**

Original databases (PubMed/MEDLINE, Web of Science, SCOPUS, and Embase) were searched using standard keywords to identify all controlled trials investigating the effects of weight loss interventions among NAFLD children and adolescents. Pooled weighted mean difference and 95% confidence intervals were achieved by random‐effects model analysis.

**Results:**

Eighteen eligible clinical trials were included in this systematic review and meta‐analysis. The pooled findings showed that especially more intense weight loss interventions significantly reduced the glucose (*p* = 0.007), insulin (*p* = 0.002), homeostatic model assessment‐insulin resistance (HOMA‐IR) (*p* = 0.003), weight (*p* = 0.025), body mass index (BMI) (*p* = 0.003), BMI *z*‐score (*p* < 0.001), waist circumference (WC) (*p* = 0.013), triglyceride (TG) (*p* = 0.001), and aspartate transaminase (AST) (*p* = 0.027). However, no significant changes were found in total cholesterol, low‐density lipoprotein cholesterol (LDL‐C), high‐density lipoprotein cholesterol (HDL‐C), alanine transaminase (ALT), and hepatic steatosis grades (all *p* > 0.05) following weight loss interventions.

**Conclusions:**

Weight loss interventions had significant effects on NAFLD‐related parameters including glucose, insulin, HOMA‐IR, weight, BMI, BMI z‐score, WC, TG, and AST.

## Graphical abstract

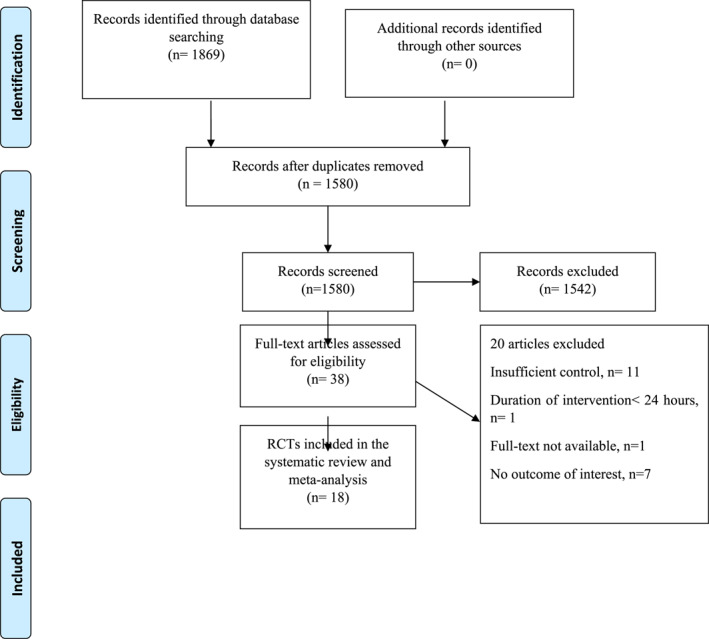

**Abbreviations**
AASLD: American Association for the Study of Liver Diseases
ALT: alanine transaminase
AST: aspartate transaminase
BMI: body mass index
CI: confidence interval
DHA: docosahexaenoic acid
D‐LMP: dietitian‐led lifestyle modification program
EPA: eicosapentaenoic acid
FDA: Food and Drug Administration
HDL‐C: high‐density lipoprotein cholesterol
HOMA: homeostatic model assessment
IGWLD: intragastric weight loss devices
IHTC: intra‐hepatic triglyceride content
IR: insulin resistance
LDL‐C: low‐density lipoprotein cholesterol
LSG: laparoscopic sleeve gastrectomy
NAFLD: non‐alcoholic fatty liver disease
NASH: non‐alcoholic steatohepatitis
NSWL: nonsurgical weight loss
P‐CON: pediatrician‐led consultation
PUFAs: polyunsaturated fatty acids
SDS: standard deviation score
TC: total cholesterol
TG: triglyceride
WC: waist circumference
WMD: weighted mean difference

## 1 INTRODUCTION

Non‐alcoholic fatty liver disease (NAFLD) is one of the main health challenges worldwide,^1^ considered the most prevalent type of chronic liver disease in children and adults,^2^ and the leading cause of adult liver transplantation.^3^ The prevalence of NAFLD has been reported as 25%–30% of the people worldwide,^4^ of which approximately 5.5%–10.3% are children.^5^ NAFLD is characterized by a broad spectrum of clinical liver abnormalities from fat deposition in more than 5% of hepatocytes, as simple hepatic steatosis (HS), to more severe and inflammatory progression including non‐alcoholic steatohepatitis ( non‐alcoholic steatohepatitis), fibrosis, cirrhosis, and even hepatocellular carcinoma^3^, ^6^, ^7^, ^8^ or end‐stage liver failure.^5^, ^6^, ^9^ Moreover, several complications, including chronic kidney disease,^10^, ^11^, ^12^ cardiovascular disease,^5^, ^7^, ^11^, ^12^, ^13^, ^14^, ^15^ and metabolic disorders, such as hypertension,^6^, ^11^, ^15^, ^16^, ^17^ dyslipidemia,^6^, ^11^, ^16^, ^17^ insulin resistance,^4^, ^5^, ^15^, ^16^ type 2 diabetes,^5^, ^7^, ^11^, ^12^, ^15^, ^17^, ^18^ and specifically obesity^4^, ^6^, ^7^, ^15^, ^16^, ^18^, are associated with not‐treated NAFLD.

Generally, the etiology of NAFLD is very complex^6^, ^19^ but refers to multifactorial causes, which can be genetic non‐modifiable factors,^20^, ^21^, ^22^ non‐genetic non‐modifiable factors (e.g., age, race, reproductive hormones, pubertal stages, and gut‐microbiome alternation),^2^ and non‐genetic modifiable factors such as environment^2^, ^23^ and lifestyle factors such as physical activity, and diet.^2^, ^24^ However, failure to observe appropriate nutritional behaviors and strategies can lead to the occurrence of metabolic risk factors such as obesity and insulin resistance, which are considered the most common causative factors of NAFLD.^4^, ^5^

The optimal current preventive and therapeutic approaches emphasize the role of nutritional supplements and formulas and the development of new drugs.^6^ Nevertheless, since there is limited existing evidence, it is not yet well‐understood which specific dietary components affect the development and progression of NAFLD in children.^5^, ^25^ Moreover, according to Food and Drug Administration, there are no approved pharmacotherapeutic agents^5^ which are targeting major molecular pathways potentially involved in the development of this disease.^6^ Several studies have shown that obesity is a strong NAFLD risk.^2^, ^3^, ^8^ Therefore, weight loss through lifestyle changes, diet, and physical activity is essential. A pediatric NAFLD cohort study showed significant improvement in liver histology and laboratory abnormalities with lifestyle interventions.^26^ However, since there are insufficient studies to determine the level required for weight loss in children with NAFLD, the ultimate goal is a weight loss of 5%–10% of baseline body weight for post‐pubertal subjects.^27^, ^28^ Although several clinical guidelines consider intensive lifestyle modification prior to starting medications,^29^ concerning the hardness of continuing long‐term weight loss through lifestyle changes in children is a challenge.

As lifestyle interventions are the main therapeutic strategy for pediatric NAFLD, there is a clear necessity for science‐based weight loss recommendations and well‐founded interventions for NAFLD patients.^5^, ^30^ Most of the previous studies assess the efficiency of such kinds of weight loss interventions on some biochemical and anthropometric parameters in children and adolescents with NAFLD in isolation and there is no review on them.^5^, ^9^, ^30^, ^31^, ^32^ Therefore, the present systematic review and meta‐analysis based on clinical trials aimed to investigate the effects of weight loss interventions on laboratory and clinical parameters in children and adolescents with NAFLD.

## 2 MATERIALS AND METHODS

The present study was documented in accordance with the PRISMA [Preferred Reporting Items for Systematic Review and Meta‐analysis] guidelines.^33^ We carried out a comprehensive systematic search in PubMed/MEDLINE, Web of Science, SCOPUS, and Embase from inception until June 2022 without using time or language restrictions. The Randomized controlled trials (RCTs) that reported the effects of weight loss interventions on weight, body mass index (BMI), BMI Z score, waist circumference (WC), alanine transaminase (ALT), Aspartate transaminase (AST), total cholesterol (TC), low‐density lipoprotein (LDL) and high‐density lipoprotein (HDL) cholesterol, triglyceride (TG), glucose, insulin, homeostatic model assessment of insulin resistance (HOMA‐IR), and HS were considered. Medical subject headings and Emtree embase were selected to search the online databases, as follows: (“non‐alcoholic fatty liver” OR “Liver Cirrhosis” OR NAFLD OR “Steatohepatit*” OR “nonalcoholic hepatic steatosis” OR “Liver Fibrosis” OR “Hepatic Cirrhosis” OR “Hepatic Fibrosis”) AND (“Weight Loss” OR “Weight Reduction Programs” OR “Obesity Management” OR “diet therapy” OR “Weight intervention” OR “weight reduce” OR “caloric restriction” OR “Anti‐Obesity Agents” OR “Antiobesity Drugs” OR “Weight Loss Drug” OR “Weight Loss Agents” OR “energy restriction” OR “Gastric Bypass“ OR “gastroplasty” OR “Bariatric Surgery” OR “gastric banding” OR “Anastomosis, Surgical” OR “Anastomosis, Roux‐en‐Y” OR “biliopancreatic diversion” OR “jejunoileal bypass”) AND (Child” OR “Adolescent” OR “Pediatrics” OR youth OR teen). Additionally, the reference lists of the articles retrieved and related review studies were also hand‐screened to fine eligible trials that might have been missed.

### 2.1 Study selection

After excluding duplicate articles, two authors independently reviewed titles, abstracts, or full text of studies to detect related articles. Finally, original studies were included in the present meta‐analysis if they had the following criteria: (1) were randomized clinical trial studies; (2) we included interventions comprising behavioral weight loss programs, pharmacotherapy, bariatric surgery, alone or in combination. Exercise or diet interventions that did not aim for weight loss were excluded. Studies in which a weight loss intervention was combined with another potential treatment for NAFLD, such as pioglitazone hydrochloride, were excluded because the effect of weight loss intervention was potentially confounded by additional effects of the medication on the pathogenesis of disease; (3) enrolled children and adolescents participants (aged <18 years); and (4) reported weight, BMI, WC, BMI‐Z score, TC, LDL and HDL cholesterol, TG, glucose, insulin, HOMA‐IR, ALT, AST, and HS as primary or secondary outcomes (5) Confirmation of NAFLD based on the criteria in children and adolescents approved by the Pediatric Gastroenterology Hepatology and Nutrition (ESPGHAN) association. The duplicated data, studies with unclear information and which did not receive any feedback from the corresponding author(s) after email, non‐randomized study designs, animal and observational studies, studies without a control group and reviews were excluded. Also, the studies that reported the duration of the intervention in hours were excluded from this study. The PICOS criteria for inclusion and exclusion of studies were as follows. Population: children and adolescents with NAFLD; Intervention: behavioral weight loss programs, pharmacotherapy, bariatric surgery, alone or in combination; Comparator: other intervention or placebo; Outcomes: weight, BMI, WC, BMI‐Z score, TC, LDL and HDL cholesterol, TG, glucose, insulin, HOMA‐IR, ALT, AST, and HS; Study design: randomized clinical trials studies.

### 2.2 Data extraction

Two independent researchers reviewed the data and an additional reviewer resolved any disagreements. The following information was abstracted: author, year of publication, country, number of intervention and control groups, participants' gender, mean age (year), duration of intervention, type of intervention or control group, and means and standard deviations of weight, BMI, WC, BMI‐Z score, TC, LDL and HDL cholesterol, TG, glucose, HbA1c, insulin, HOMA‐IR, ALT, AST, and hepatic steatosis at baseline, post‐treatment and/or changes between baseline and post‐treatment.

### 2.3 Quality assessment

The Cochrane Risk of Bias Tool for RCTs^34^ was used by two authors to identify potential risks of bias. The quality assessment tool encompasses the following items: adequacy of random sequence generation, allocation concealment, blinding, and the detection of incomplete outcome data as well as selective outcome reporting, and other potential sources of bias. Based on the recommendations of the Cochrane Handbook, judgment of each domain was recorded as “Low”, “High”, or “Unclear” risk of bias. Any disagreement in the data extraction and the risk of bias assessment was resolved by a third reviewer. Also, Grading of Recommendations Assessment, Development, and Evaluation (GRADE) scoring system was used to evaluate the quality of the current analysis study.^35^ The GRADE checklist is a valid 10‐point scoring system that measures factors influencing study quality. This scale includes seven items: (1) risk of bias, study quality, and study limitations, (2) precision, (3) heterogeneity, (4) directness, (5) publication bias, (6) funding bias, (7) study design.

### 2.4 Data synthesis and statistical analysis

The statistical analysis was performed using RevMan V.5.3 software and STATA version 12.0 (Stata Corp, College Station). In addition, Endnote software was used to remove duplicate articles and manage eligible articles. If data were expressed in a different format, standard calculations were executed to obtain the mean and SD.^36^, ^37^ For instance, if the SDs of the change were not stated in the trials, we derived it using the following formula: SD changes = square root [(SD baseline ^2^ + SD final ^2^)–(2 × R × SD baseline × SD final)]. Also, for trials that only reported standard error of the mean (SEM), SDs were obtained using the following formula: SD = SEM × √*n*, where “*n*” is the number of subjects in each group. The random‐effects model was used for the meta‐analysis of study outcomes. The weighting of studies was done using the generic inverse variance method. In case of multiple evaluations in a single study group, the values belonging to the longest time point were used for the analyses. Heterogeneity was examined using the I‐squared (*I* ^2^) statistic, in which the source of heterogeneity was determined if the *I* ^2^ value was >50%, or if there in the case of inconsistency across RCT data.^38^ In order to identify potential sources of heterogeneity, a pre‐defined subgroup analysis based on the duration of intervention, and type of intervention was performed. A sensitivity analysis was performed to assess the contribution of each study to the overall mean difference. We assessed the presence of publication bias using the formal Egger's test.^39^

## 3 RESULTS

**FIGURE 1:**
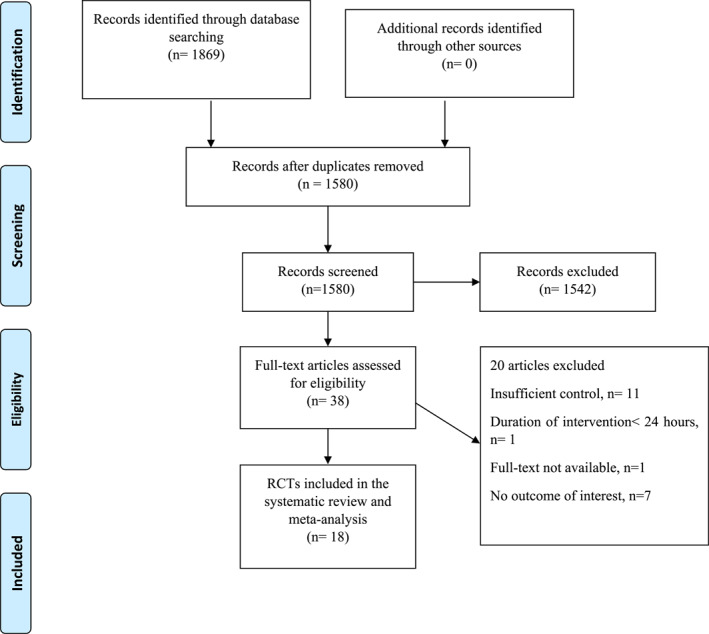
Flowchart of the study.

Figure 1 shows a flowchart of the study selection process and reasons for excluding articles. Then, 1869 publications from the aforementioned electronic databases are yielded in this figure. After excluding duplicate studies, a total of 1580 publications remained. Then, we reviewed the title/abstract of studies, and excluded articles which did not meet the inclusion criteria. 38 articles were retrieved during the secondary screening by full‐text. Of those, 20 studies were discarded for the different reasons. Finally, 18 studies met the eligibility criteria and were included in the quantitative meta‐analysis.^25^, ^26^, ^30^, ^32^, ^40^, ^41^, ^42^, ^43^, ^44^, ^45^, ^46^, ^47^, ^48^, ^49^, ^50^, ^51^, ^52^, ^53^

### 3.1 Study characteristics

Characteristics of the pooled studies are presented in Table 1. Six studies were conducted in Italy, three articles in the USA, and six studies were conducted in Iran, China and Germany. Other RCT were performed in Brazil, Poland, and Turkey. All articles were published between 2006 and–2021. All of the included studies were RCTs and the duration of the studies ranged from 4 to 96 weeks. The age of the participants ranged from 7.4 to 15.69 years and the percentage of participants in the studies with male gender varied from 33 to 100. Based on the type of intervention, one study using bariatric surgery, 4 studies with a combination of diet and exercise, one article with diet restriction alone, 5 studies using a combination of metformin with exercise and diet, one study with intervention carnitine, 2 studies were also conducted with the combination of omega‐3 with weight loss interventions and the rest of the studies were also conducted with the combination of vitamin E with other weight loss interventions.

**TABLE 1:** Include randomized controlled trial study characteristics by population.

Study ID	Country	Age	Sex (% male)	Sample size (intervention)	Sample size (control)	Intervention group	Control group	Duration (week)	Outcomes
Manco et al. 2017	Italy	15/69	43/5	20	53	Laparoscopic sleeve gastrectomy (LSG)	Nonsurgical weight loss (NSWL)	48	FBS, Insulin, HOMA‐IR, HDL, LDL, TG, TC, weight, BMI‐Z score, BMI, WC, ALT, AST
Chan et al. 2018	China	14/5	63	26	26	Diet and Exercise	Usual care	16	FBS, Insulin, HOMA‐IR, HDL, LDL, TG, TC, weight, BMI‐Z score, BMI, WC, ALT, AST
Reinehr et al. 2008	Germany	10/5	45	21	15	Diet restriction	Usual care	48	FBS, Insulin, HOMA‐IR, HDL, LDL, TG, HC, BMI‐Z score, BMI, WC, ALT, AST
Wang et al. 2008	China	13/7	68	19	38	Diet and Exercise	Usual care	4	FBS, Insulin, HOMA‐IR, TG, TC, BMI‐Z score, BMI, ALT, AST
Nobili et al. 2008	Italy	12	71	28	29	Diet and Exercise and 1.5 gr metformin	Diet and Exercise and placebo	96	FBS, Insulin, HOMA‐IR, TG, TC, BMI, ALT, AST
Lavine et al. 2011	USA	13/1	81	57	58	Diet + Exercise and 1gr metformin	Diet + placebo	96	FBS, HOMA‐IR, HDL, LDL, TG, TC, HS, weight, BMI‐Z score, BMI, WC, ALT, AST
Tock et al. 2010	Brazil	NR	NR	21	14	Diet and Exercise and 1 gr metformin	Diet and Exercise and placebo	48	FBS, Insulin, HOMA‐IR, weight, BMI, ALT, AST
Nadeau et al. 2009	USA	15/1	33	37	13	Diet and Exercise and 1/7 gr metformin	Diet and Exercise and placebo	24	FBS, Insulin, HDL, LDL, TG, TC, BMI, ALT, AST
Saneian et al. 2021	Iran	12/3	76	30	25	Diet and Exercise and 1 gr carnitine	Diet and Exercise and placebo	12	weight, BMI‐Z score, BMI, WC, ALT, AST
Ghergherehchi et al. 2013	Iran	7/4	84/8	17	16	Diet and Exercise and 400 mg Vit E	Diet and Exercise and placebo	24	LDL, TG, TC, BMI, ALT, AST
Nobili et al. 2006	Italy	13/07	46	45	43	Diet and Exercise and vitamin E 600 IU/day and vitamin C 500 mg/day	Diet and Exercise and placebo	48	FBS, Insulin, HOMA‐IR, TG, TC, weight, BMI, ALT, AST
Nobili et al. 2008	Italy	13/07	46	45	43	Diet and Exercise and vitamin E 600 IU/day and vitamin C 500 mg/day	Diet and Exercise and placebo	48	FBS, HOMA‐IR, TG, TC, weight, BMI, ALT, AST
Pacifico et al. 2015	Italy	11	58/5	25	26	Diet and Exercise and DHA supplementation (250 mg/day)	Diet and Exercise and placebo	24	FBS, Insulin, HOMA‐IR, HDL, TG, TC, weight, BMI‐Z score, BMI, WC, ALT
Akcam et al. 2011	Turkey	12/3	47/7	22	22	Diet and Exercise and 850‐mg metformin	Diet and Exercise and placebo	24	FBS, Insulin, HOMA‐IR, TG, BMI‐Z score, BMI
B. Schwimmer et al. 2019	USA	13/1	100	20	20	Diet low in free sugars	Usual Diet	8	FBS, Insulin, HOMA‐IR, HDL, LDL, TG, TC, HC, ALT
Janczyk et al. 2015	Poland	13	86	30	34	Diet and Exercise and omega‐3 fatty acids (docosahexaenoic acid and eicosapentaenoic acid, 450–1300 mg/day)	Diet and Exercise and omega‐6 sunflower oil	24	FBS, Insulin, HOMA‐IR, HDL, LDL, TG, TC, BMI‐Z score, ALT, AST
Vajro et al. 2004	Italy	10	78	14	14	Diet and Exercise and 400 mg Vit E	Diet and Exercise and placebo	8	ALT
Reinehr et al. 2009	Germany	NR	57	109	43	Diet + exercise	Usual care	96	BMI‐*Z* score

Abbreviations: ALT, alanine transaminase; AST, aspartate transaminase; BMI, body mass index; FBS, fasting blood sugar; HDL, high‐density lipoproteins; HOMA‐IR, Homeostatic Model Assessment for Insulin Resistance; HS, hepatic steatosis; IU, international unit; LDL, low‐density lipoproteins; NR, not reported; RCT, randomized controlled trial; TC, total cholesterol; WC, waist circumferences.

Table 2 showed the results of the quality assessment of eligible studies. Also, after evaluating the quality of the present meta‐analysis based on the GRADE score system, a score of 8.6 (very good quality) was calculated.

**TABLE 2:** Risk of bias assessment according to the Cochrane collaboration’s risk of bias assessment tool.

Study, year (reference)	Random sequence generation	Allocation concealmen	Blinding of participants and personnel	Blinding of outcome assessment	Incomplete outcomedata	Selective reporting	Overall assessment of risk of bias
Manco et al. 2017	Unclear	Unclear	Unclear	Unclear	Low	Low	Unclear
Chan et al. 2018	Low	Unclear	Unclear	Low	Low	Low	Unclear
Reinehr et al. 2008	Unclear	Unclear	Unclear	Unclear	Unclear	Unclear	Unclear
Wang et al. 2008	Low	Unclear	Unclear	Unclear	Low	Low	Unclear
Nobili et al. 2008	Low	Unclear	Low	Low	Low	Unclear	Unclear
Lavine et al. 2011	Low	Low	Unclear	Low	Low	Low	Unclear
Tock et al. 2010	Unclear	Unclear	Unclear	Unclear	Low	Low	Unclear
Nadeau et al. 2009	Unclear	Unclear	Unclear	Unclear	Unclear	Unclear	Unclear
Saneian et al. 2021	Low	Low	Unclear	Low	Unclear	Low	Unclear
Ghergherehchi et al. 2013	Unclear	Unclear	Unclear	Unclear	Unclear	Unclear	Unclear
Nobili et al. 2006	Low	Unclear	Unclear	Unclear	Unclear	Unclear	Unclear
Nobili et al. 2008	Low	Low	Low	Low	Low	Unclear	Low
Pacifico et al. 2015	Unclear	Unclear	Unclear	Unclear	Low	Low	Unclear
Akcam et al. 2011	Unclear	Unclear	Unclear	Unclear	Unclear	Unclear	Unclear
B. Schwimmer et al. 2019	Low	Unclear	Unclear	Unclear	Low	Low	Unclear
Janczyk et al. 2015	Low	Unclear	Low	Low	Low	Unclear	Unclear
Vajro et al. 2004	Low	Unclear	Low	Low	Low	Low	Low
Reinehr et al. 2009	Low	Unclear	Unclear	Unclear	Unclear	Unclear	Unclear

## 4 META‐ANALYSIS

### 4.1 Effects of weight loss interventions on glycemic parameters

**FIGURE 2:**
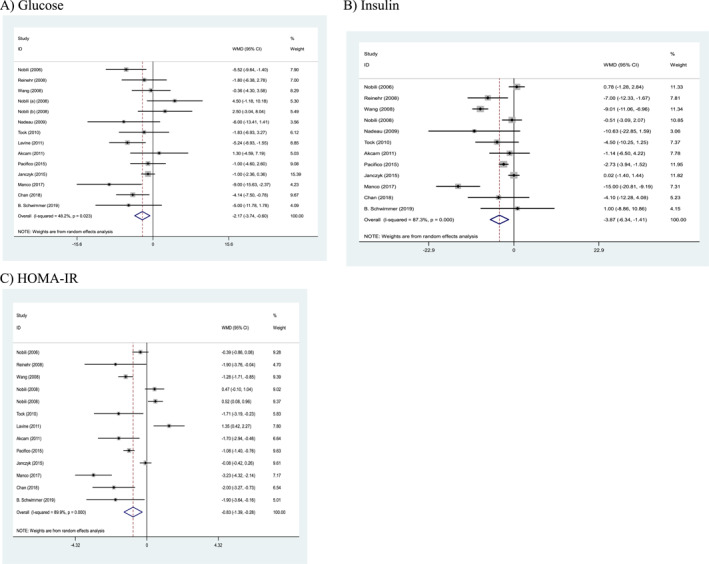
Forest plots from the meta‐analysis of clinical trials investigating the effects of weight loss interventions on (A) glucose, (B) insulin, and (C) homeostatic model assessment‐insulin resistance (HOMA‐IR). HOMA‐IR, homeostatic model assessment‐insulin resistance; WMD, weighted mean difference.

Our findings showed that more intense weight loss interventions compared to those with less intensity, significantly reduced glucose (weighted mean difference (WMD): −2.17 mg/dL, 95% (confidence interval) CI: −3.74 to −0.60, *p* = 0.007), insulin (WMD: −3.87 μU/mL, 95% CI: −6.34 to −1.41, *p* = 0.002), and homeostatic model assessment (HOMA)‐IR (WMD: −0.83, 95% CI: −1.39 to −0.28, *p* = 0.003). High heterogeneity was shown in the trials for glucose (Cochran's *Q* test, *p* = 0.023, *I* ^2^ = 48.2%), insulin (Cochran's *Q* test, *p* < 0.001, *I* ^2^ = 87.3%), and HOMA‐IR (Cochran's *Q* test, *p* < 0.001, *I* ^2^ = 89.9%; Figure 2).

### 4.2 Effects of weight loss interventions on anthropometric parameters

**FIGURE 3:**
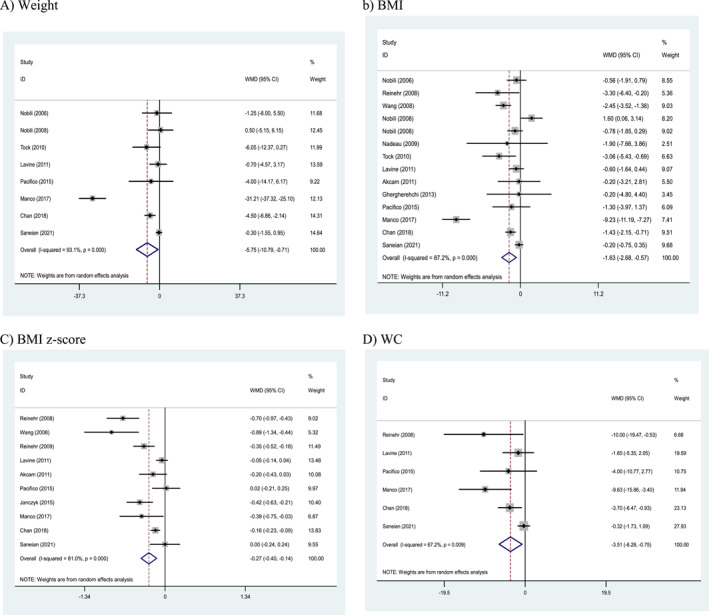
Forest plots from the meta‐analysis of clinical trials investigating the effects of weight loss interventions on (A) weight, (B) BMI, (C) body mass index (BMI) *z*‐score, and (D) waist circumference (WC). BMI, Body mass index; WC, Waist circumference; WMD, weighted mean difference.

Compared to control groups, the effect of more intense weight loss interventions on anthropometric parameters showed a significant difference for all parameters including weight (WMD: −5.75 kg, 95% CI: −10.79 to −0.71, *p* = 0.025), body mass index (BMI) (WMD: −1.63 kg/m^2^, 95% CI: −2.68 to −0.57, *p* = 0.003), BMI *z*‐score (WMD: −0.27, 95% CI: −0.40 to −0.14, *p* < 0.001), and WC (WMD: −3.51 cm, 95% CI: −6.28 to −0.75, *p* = 0.013). There was evidence of significant between‐study heterogeneity (Cochran's *Q* test, *p* < 0.001, *I* ^2^ = 93.1% for weight; Cochran's *Q* test, *p* < 0.001, *I* ^2^ = 87.2% for BMI; Cochran's *Q* test, *p* < 0.001, *I* ^2^ = 81.0% for BMI *z*‐score; Cochran's *Q* test, *p* = 0.009, *I* ^2^ = 67.2% for WC) in all meta‐analyses (Figure 3).

### 4.3 Effects of weight loss interventions on lipid parameters

**FIGURE 4:**
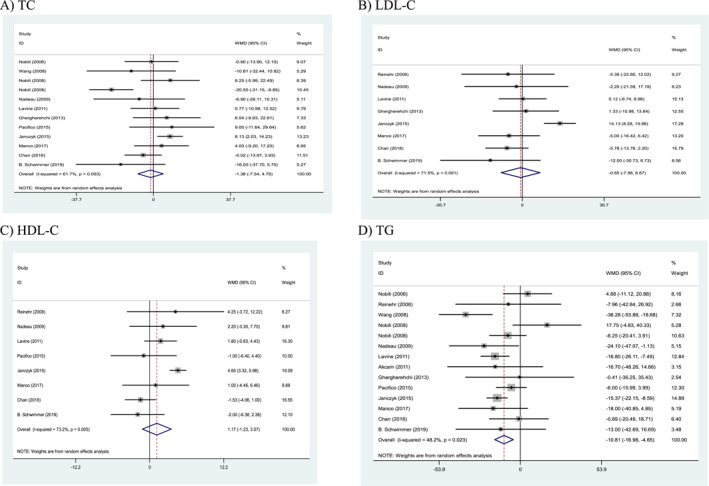
Forest plots from the meta‐analysis of clinical trials investigating the effects of weight loss interventions on (A) total cholesterol (TC), (B) low‐density lipoprotein cholesterol (LDL‐C), (C) high‐density lipoprotein (HDL), and (D) triglyceride (TG). HDL‐C, high‐density lipoprotein cholesterol; LDL‐C, low‐density lipoprotein cholesterol; TC, total cholesterol; TG, triglyceride; WMD, weighted mean difference.

The results of the combined data showed a significant effect on the levels of triglyceride (TG) (WMD: −10.81 mg/dL, 95% CI: −16.98 to −4.65, *p* = 0.001) following more intense weight loss interventions. However, no significant effect was observed on the levels of other lipid parameters after the intervention) WMD: −1.38 mg/dL, 95% CI: −7.54 to 4.78, *p* = 0.660 for TC; WMD: −0.65 mg/dL, 95% CI: −7.98 to 6.67, *p* = 0.862 for LDL cholesterol (LDL‐C); WMD: 1.17 mg/dL, 95% CI: −1.23 to 3.57, *p* = 0.340 for high‐density lipoprotein cholesterol (HDL‐C)). Furthermore, a significant heterogeneity was observed between these trials for TC (Cochran's *Q* test, *p* = 0.003, *I* ^2^ = 61.7%), TG (Cochran's *Q* test, *p* = 0.023, *I* ^2^ = 48.2%), HDL‐C (Cochran's *Q* test, *p* < 0.001, *I* ^2^ = 73.2%), and LDL‐C levels (Cochran's *Q* test, *p* = 0.001, *I* ^2^ = 71.5%; Figure 4).

### 4.4 Effects of weight loss interventions on liver parameters

**FIGURE 5:**
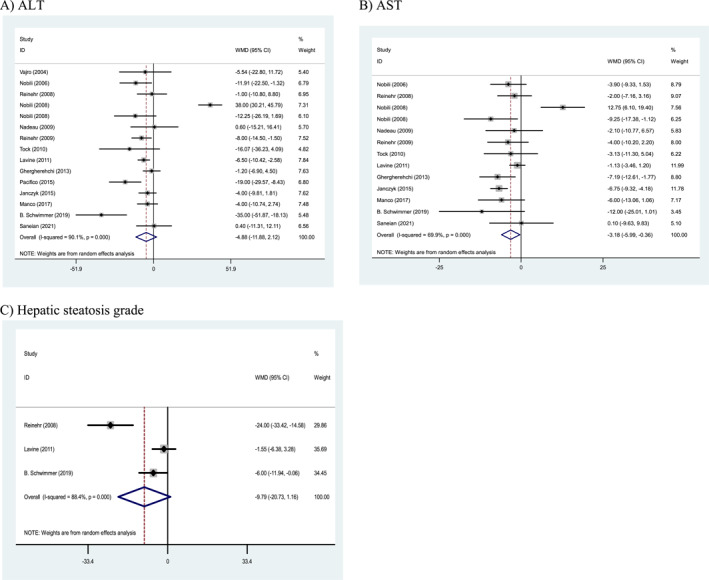
Forest plots from the meta‐analysis of clinical trials investigating the effects of weight loss interventions on (A) alanine transaminase (ALT), (B) aspartate transaminase (AST), and (C) hepatic steatosis (HS) grade. ALT, alanine transaminase; AST, aspartate transaminase; WMD, weighted mean difference.

Pooled results from the random‐effects model indicated that ALT level (WMD: −4.88, U/L, 95% CI: −11.88 to 2.12, *p* = 0.172) and hepatic steatosis grade (WMD: −9.79 U/L, 95% CI: −20.73 to 1.16, *p* = 0.080) did not change significantly following more intense weight loss interventions. However, more intense weight loss interventions significantly improved AST level (WMD: −3.18, U/L, 95% CI: −5.99 to −0.36, *p* = 0.027) compared to the control group. Also, a high heterogeneity being noted among the studies for ALT (Cochran's *Q* test, *p* < 0.001, *I* ^2^ = 91.0%), AST (Cochran's *Q* test, *p* < 0.001, *I* ^2^ = 69.9%), and hepatic steatosis grade (Cochran's *Q* test, *p* < 0.001, *I* ^2^ = 88.4%; Figure 5).

### 4.5 Subgroup analysis

Subgroup results showed that more intense weight loss interventions led to greater reductions in glucose, HOMA‐IR, BMI, TG, ALT, and AST over a period equal to or less than 24 weeks. However, this effect was observed for BMI *z*‐score and WC during the intervention for more than 24 weeks. Moreover, the findings showed that intervention with diet, exercise, and vitamin E had a greater effect on ALT and AST levels compared with other weight loss interventions. On the other hand, bariatric surgery seems to have a more significant effect on the levels of insulin, glucose, and BMI. However, only one study has been conducted on bariatric surgery and the certainty of this finding cannot be stated (Figures S1).

### 4.6 Sensitivity analysis

We removed each study from the analysis step by step to discover the impact of each single trial on the pooled effect size for the levels of glucose, insulin, HOMA‐IR, weight, BMI, BMI z‐score, WC, TG, LDL‐C, HDL‐C, TC, ALT, AST, and the grade of hepatic steatosis. The leave‐one‐out sensitivity analysis showed robustness of the findings (Figures S1).

### 4.7 Publication bias

Evaluation of publication bias by visual inspection of funnel plot and Egger's test demonstrated no evidence for publication bias in the meta‐analysis of weight loss interventions on the levels of glucose (*p* = 0.702), insulin (*p* = 0.411), HOMA‐IR (*p* = 0.903), weight (*p* = 0.458), BMI (*p* = 0.352), BMI *z*‐score (*p* = 0.180), WC (*p* = 0.188), TG (*p* = 0.477), LDL‐C (*p* = 0.805), HDL‐C (*p* = 0.216), TC (*p* = 0.784), ALT (*p* = 0.729), AST (*p* = 0.903), and the grade of hepatic steatosis (*p* = 0.117; Figure S1).

## 5 DISCUSSION

The results of the present study indicated that more intense weight loss interventions significantly improved the glucose, insulin, HOMA‐IR, weight, BMI, BMI *z*‐score, WC, TG, and AST in the intervention group compared with the control group. However, no significant differences were found in TC, LDL‐C, HDL‐C, ALT, and hepatic steatosis grades. Subgroup analyses also showed that most parameters were affected, especially by intensity, duration, and type of weight loss interventions.

In the present systematic review and meta‐analysis study, eligible clinical trials evaluated the effects of bariatric surgery,^54^ diet,^25^, ^40^ diet and exercise,^30^, ^41^, ^55^ diet and exercise, and metformin supplement,^42^, ^43^, ^44^, ^45^, ^53^ diet and exercise, and carnitine supplement,^46^ diet and exercise, and vitamin E supplement,^26^, ^47^, ^50^, ^51^ diet and exercise, and omega‐3 supplement^49^, ^52^ in children and adolescents with NAFLD. However, no studies investigated the effects of exercise or any supplements as the sole intervention.

According to our results, glucose, insulin, and HOMA‐IR were significantly lower among NAFLD children and adolescents after more intense weight loss, especially equal to or less than 24 weeks interventions. Some studies investigated the association between glycemic parameters with weight loss interventions for more than 24 weeks^26^, ^40^, ^42^, ^43^, ^45^, ^50^, ^54^ and equal to or less than 24 weeks^25^, ^30^, ^41^, ^44^, ^49^, ^52^, ^53^ in pediatric NAFLD.

Our findings are generally in contrast or in line with some of the studies selected for this review. Janczyk et al.^52^ found no differences in the levels of fasting glucose and insulin and HOMA‐IR between those who received omega‐3 fatty acids and those who received a placebo. It has been frequently reported that long‐chain polyunsaturated fatty acids (PUFAs), especially the omega‐3 fatty acids eicosapentaenoic acid (EPA) and docosahexaenoic acid (docosahexaenoic acid (DHA)) decrease in NAFLD patients.^56^ Increased levels of omega‐3 enable fat metabolism to shift away from hepatic de novo lipogenesis and toward oxidation and secretion of fatty acids.^56^, ^57^ However, there are contradictory results on the beneficial effects of omega‐3 on NAFLD,^31^, ^58^ and its overall impact on NAFLD mainly remains unknown.^57^ It is probable that its effects depend on the dose and duration of supplementation, EPA to DHA ratio, and patient‐specific factors.^59^

Over the last decade, the increased rates of obesity have led to the increased prevalence of NAFLD.^6^, ^7^, ^14^, ^60^ Given the high importance of obesity as the most common risk factor for developing NAFLD, clinical management primarily emphasizes weight loss. Bariatric surgery, including laparoscopic sleeve gastrectomy (LSG), is recognized as a therapeutic option that achieves long‐term weight reduction.^61^, ^62^ Manco et al.^54^ found that those who underwent LSG had more reduction of glucose‐120 mg/L, insulin, and HOMA‐IR without differences in fasting glucose compared to those who received lifestyle intervention plus intragastric weight loss devices (intragastric weight loss devices (IGWLD)) and those who received only lifestyle intervention with nonsurgical weight loss (nonsurgical weight loss (NSWL)).

Similarly, Reinehr et al.^40^ found a negative association between substantial weight loss through lifestyle intervention “Obeldicks” (consisting of physical activity, behavioral, and dietary plans) with insulin and HOMA‐IR and no association with glucose. In line with many recommendations that emphasized the reduction of calorie and carbohydrate intake, B. Schwimmer et al.^25^ reported that there were more reductions in the level of glucose and no differences in insulin and HOMA‐IR after a diet low in free sugars.

Regarding anthropometric parameters, weight, BMI, BMI *z*‐score, and WC were significantly lower among NAFLD children and adolescents after more intense weight loss, especially equal to or less than 24 weeks (for weight and BMI) and more than 24 weeks (for BMI *z*‐score and WC) interventions. Some studies investigated the association between anthropometric parameters with weight loss interventions for more than 24 weeks^26^, ^40^, ^42^, ^43^, ^45^, ^50^, ^54^, ^55^ and equal to or less than 24 weeks^30^, ^41^, ^44^, ^46^, ^47^, ^49^, ^51^, ^52^, ^53^ in pediatric NAFLD. Continued abnormal weight gain is a common cause of NAFLD development.^9^ Interestingly, inefficient regulation of fat metabolism in the liver may lead to fat deposition around the abdominal area and then weight gain.^63^ So, what is effective is the correct dietary composition to improve liver fat‐burning function.^64^

Nobili et al.^50^ found that there were no differences in weight loss either with a balanced calorie diet, physical activity, and placebo or with a vitamin E plus vitamin C. Another study conducted by Vajro et al.^51^ reported no differences in the levels of weight loss with vitamin E or placebo. Also, there were differences in the level of weight loss between only diet and only vitamin E supplements. Moreover, Wang et al.^41^ demonstrated that both simple lifestyle intervention and vitamin E improved BMI and BMI *z*‐score. Previous studies reported that both vitamin E and metformin improved body mass index standard deviation score^53^ and there was an improvement and minor changes in BMI after both vitamin E and placebo.^47^ Similarly, Nobili et al.^26^ found that each vitamin E plus vitamin C and placebo improved BMI and weight. Also, Chan et al.^30^ found that both D‐LMP and P‐CON intervention reduced weight, BMI, BMI *z*‐score, and WC.

Hepatic lipid homeostasis is under the control of precise interactions that any disruption of them may accelerate the overload of intrahepatocellular lipid and the progress of NAFLD.^65^ The dysregulation of lipid homeostasis in NAFLD is characterized by decreased fatty acid oxidation, increased hepatic lipid uptake and de novo lipogenesis, extreme production and secretion of very LDL, and impaired HDL‐mediated cholesterol efflux.^66^ However, the exact molecular mechanisms of pathological liver fat accumulation remain widely unknown.^65^, ^67^ According to the results of the current study, TG was significantly lower among NAFLD children and adolescents after more intense weight loss, especially equal to or less than 24 weeks, interventions. However, no significant differences were found in TC, LDL‐C, and HDL‐C for equal to or less than 24 weeks or more than 24 weeks of intervention. Some studies investigated the association between lipid parameters with weight loss interventions for more than 24 weeks^26^, ^40^, ^42^, ^45^, ^54^ and equal to or less than 24 weeks^25^, ^30^, ^41^, ^44^, ^47^, ^49^, ^51^, ^52^, ^53^ in pediatric NAFLD.

Manco et al.^54^ found that there were no differences in TG, LDL‐C, HDL‐C, and TC after intervention with LSG, IGWLD, or NSWL. Similarly, Janczyk et al.^52^ found that there were no differences in the levels of TG, LDL‐C, HDL‐C, and TC after intervention with omega‐3 fatty acids or placebo. Moreover, another study^30^ found that there were no differences in LDL‐C, HDL‐C, or TG after D‐LMP or P‐CON. However, both interventions reduced intra‐hepatic triglyceride content. Also, Reinehr et al.^40^ found no association between “Obeldicks” with TG and HDL‐C. Another study conducted by Wang et al.^41^ reported that both simple lifestyle intervention and vitamin E improved TG and TC. Consistent with the previous study, Nobili et al.^42^ found that both metformin and placebo improved TG and TC. Also, Akcam et al.^53^ found that both vitamin E and metformin improved TG. Similarly, another study^26^ found that both vitamin E plus vitamin C receivers and placebo receivers had an improvement without differences in TG.

Aspartate transaminase and ALT are liver aminotransferase enzymes that widely exist in the liver cytosol. Alanine transaminase serum level as a standard biomarker of liver function is commonly used to mirror liver damage in NAFLD patients.^68^, ^69^ Our results showed that AST was significantly lower among NAFLD children and adolescents after more intense weight loss, especially equal to or less than 24 weeks, interventions. However, there were no significant differences in ALT levels and hepatic steatosis grade for equal to or less than 24 weeks or more than 24 weeks of intervention. Some studies investigated the association between liver parameters with weight loss interventions for more than 24 weeks^26^, ^40^, ^42^, ^43^, ^45^, ^50^, ^54^, ^55^ and equal to or less than 24 weeks^25^, ^30^, ^41^, ^44^, ^46^, ^47^, ^49^, ^51^, ^52^ in pediatric NAFLD.

Chan et al.^30^ found that there were no differences in ALT and AST after each D‐LMP or P‐CON intervention. Moreover, Reinehr et al.^40^ showed no association between “Obeldicks” with AST and ALT. Consistent with previous studies, Nobili et al.^50^ found that there were no differences in ALT either with a balanced calorie diet, physical activity, and placebo or with a vitamin E plus vitamin C. However, Wang et al.^41^ found that both simple lifestyle interventions with exercise and a low‐calorie diet and vitamin E improved ALT and AST. Another study conducted by Nadeau et al.^44^ reported that both metformin and placebo improved ALT. However, neither of them changed AST. While another study^42^ found that both metformin and placebo improved ALT and AST levels, hepatic steatosis grade, and NAFLD activity score. Similarly, Nobili et al.^26^ found that both vitamin E plus vitamin C receivers and placebo receivers showed improvements without differences in ALT and AST levels and NAFLD activity score. In contrast with previous studies, Lavine et al.^45^ demonstrated that both vitamin E and metformin similarly reduced ALT. However, neither vitamin E nor metformin improved ALT and AST levels or hepatic steatosis grade. However, Saneian et al.^46^ found that both L‐carnitine and placebo improved AST and ALT without a reduction in NAFLD grade. Moreover, no differences were found in AST and ALT changes.

On the other hand, Manco et al.^54^ found that those who underwent LSG had a greater reduction in ALT. However, there were no differences in AST after each intervention. Another study conducted by B. Schwimmer et al.^25^ reported that a diet low in free sugars reduced ALT and AST levels and hepatic steatosis grades more than a regular diet. Consistent with previous studies, Reinehr et al. and Tock et al. found that “Obeldicks”^55^ and metformin^43^ reduced ALT and AST. Moreover, Ghergherehchi et al.^47^ found an improvement and only minor changes in ALT and AST after both vitamin E and placebo. Also, hepatic steatosis grade was similar after both. However, Janczyk et al.^52^ revealed that omega‐3 fatty acids reduced AST. However, there was no improvement in hepatic steatosis grade and no difference in ALT. Also, another study^51^ found that changes in ALT levels were lower in only diet compliers. However, no differences were found in the levels of ALT. Similarly, Pacifico et al.^49^ found that DHA receivers had a reduction in ALT. However, neither DHA nor placebo receivers had differences in ALT levels.

### 5.1 Strengths and limitations

The present study has several major strengths. First, this is the first systematic review and meta‐analysis investigating the impact of weight loss interventions among children and adolescents with NAFLD. Second, the causal inference of our results is strong due to the design of the meta‐analysis based on eligible clinical trials. Third, we considered the Cochrane Bias Methods to minimize systematic errors and achieve reliable estimates of effects. Last but not least, the results of our study may contribute to determining ways in which specialists can make more informed decisions on which types of weight loss interventions through lifestyle modifications are most appropriate in achieving constant NAFLD improvement.

However, our study had some limitations that limited the extraction of robust conclusions. Clinically and statistically significant heterogeneities were found. These may be explained by the differences in the intervention‐specific factors (e.g., type of regimen, doses of supplements, and duration of protocols), patient‐specific factors (e.g., genes, age, sex, ethnicity, and any history of the disease, drug or supplement consumption, and substance allergies), and NAFLD‐specific factors (e.g., baseline severity and its methods of screening and diagnosis). Nonetheless, we attempted to identify some possible sources of heterogeneity in data by performing a subgroup analysis.

## 6 CONCLUSIONS

In general, the present meta‐analysis demonstrated that weight loss interventions may be able to significantly improve glycemic parameters, TG, and AST levels and are associated with a trend toward reduced anthropometric parameters in children and adolescents with NAFLD. The beneficial effect seemed greatest in those trials with more intense weight loss interventions. Continuous and multidimensional lifestyle intervention for NAFLD patients might optimize the therapeutic effect of weight loss. Further homogeneous and well‐powered clinical trials on the appropriate type and duration of personalized treatment strategies are required and should aim at evaluating how weight loss interventions can improve the NAFLD and discovering the underlying mechanisms in both young and old ages. While we expect new prospective studies that allow us to tailor such interventions, we should not ignore the wealth of evidence already documented on the effects of weight loss interventions on NAFLD that has the potential to assist scholars in dealing with this epidemic.

## AUTHOR CONTRIBUTIONS

Mohammad Hassan Sohouli and Seyedeh Bagheri contributed to the conception, design, and statistical analysis. Seyedeh Bagheri, Pejman Rohani, and Mohammad Hassan Sohouli contributed to data collection and manuscript draft. Pejman Rohani, Mohammad Hassan Sohouli, and Seyedeh Bagheri supervised the study. Somaye Fatahi contributed to the manuscript draft and critical revision. All authors approved the final version of the manuscript.

## CONFLICT OF INTEREST STATEMENT

The authors declare that they have no competing interests.

## Supporting information

Supporting Information S1

Supporting Information S2

Supporting Information S3

## Data Availability

Data available on request due to privacy/ethical restrictions.
