# Supplementary material for: The effects of weight loss interventions on children and adolescents with non‐alcoholic fatty liver disease: A systematic review and meta‐analysis
Source: Obes Sci Pract. 2024 Apr 26;10(3):e758. doi: 10.1002/osp4.758 (PMC11047132; doi:10.1002/osp4.758)
Supplement: Supplementary file 2 — Supporting Information S2 [file OSP4-10-e758-s001.docx]

| A) Glucose   | B) Insulin   |
| --- | --- |
| C) HOMA-IR   |  |
| **Figure S5.** Sensitivity analysis of the weighted mean difference (*WMD*) for (A) glucose, (B) insulin, and (C) homeostatic model assessment-insulin resistance (*HOMA-IR*) | |

| A) Weight   | B) BMI   |
| --- | --- |
| C) BMI-z-score   | D) WC   |
| **Figure S6.** Sensitivity analysis of the weighted mean difference (*WMD*) for (A) weight, (B) body mass index (*BMI*), (C) BMI z-score, and (D) waist circumference (*WC*) | |

| A) CT   | B) LDL-C   |
| --- | --- |
| C) HDL-C   | D) TG   |
| **Figure S7.** Sensitivity analysis of the weighted mean difference (*WMD*) for (A) total cholesterol (*CT*), (B) low-density lipoprotein cholesterol (*LDL-C*), (C) high-density lipoprotein cholesterol (*HDL-C*), and (D) triglyceride (*TG*) | |

| 1. ALT    | B) AST   |
| --- | --- |
| C) Hepatic steatosis grade   |  |
| **Figure S8.** Sensitivity analysis of the weighted mean difference (*WMD*) for (A) alanine transaminase (*ALT*), (B) aspartate transaminase (*AST*), and (C) hepatic steatosis grade | |
