# Supplementary material for: The effects of weight loss interventions on children and adolescents with non‐alcoholic fatty liver disease: A systematic review and meta‐analysis
Source: Obes Sci Pract. 2024 Apr 26;10(3):e758. doi: 10.1002/osp4.758 (PMC11047132; doi:10.1002/osp4.758)
Supplement: Supplementary file 3 — Supporting Information S3 [file OSP4-10-e758-s002.docx]

| A) Duration   | B) Type   |
| --- | --- |
| **Figure S9*.*** Forest plots from the meta‐analysis of clinical trials investigating the effects of (A) duration (week) and (B) type of weight loss interventions on glucose. *WMD*: weighted mean difference | |

| A) Duration   | B) Type   |
| --- | --- |
| **Figure S10.** Forest plots from the meta‐analysis of clinical trials investigating the effects of (A) duration (week) and (B) type of weight loss interventions on insulin. *WMD*: weighted mean difference | |

| A) Duration   |
| --- |
| **Figure S11.** Forest plots from the meta‐analysis of clinical trials investigating the effects of (A) duration (week) of weight loss interventions on HOMA-IR. *WMD*: weighted mean difference, *HOMA-IR*: Homeostatic model assessment-insulin resistance |

| A) Duration   |
| --- |
| **Figure S12.** Forest plots from the meta‐analysis of clinical trials investigating the effects of (A) duration (week) of weight loss interventions on weight. *WMD*: weighted mean difference |

| A) Duration   | B) Type   |
| --- | --- |
| **Figure S13.** Forest plots from the meta‐analysis of clinical trials investigating the effects of (A) duration (week) and (B) type of weight loss interventions on BMI. *WMD*: weighted mean difference, *BMI*: Body mass index | |

| A) Duration   |
| --- |
| **Figure S14.** Forest plots from the meta‐analysis of clinical trials investigating the effects of (A) duration (week) of weight loss interventions on BMI z-score. *WMD*: weighted mean difference, *BMI*: Body mass index |
| A) Duration   |
| **Figure S15.** Forest plots from the meta‐analysis of clinical trials investigating the effects of (A) duration (week) of weight loss interventions on WC. *WMD*: weighted mean difference, *WC*: Waist circumference |

| A) Duration   | B) Type   |
| --- | --- |
| **Figure S16.** Forest plots from the meta‐analysis of clinical trials investigating the effects of (A) duration (week) and (B) type of weight loss interventions on TG. *WMD*: weighted mean difference, *TG*: Triglyceride | |

| A) Duration   |
| --- |
| **Figure S17.** Forest plots from the meta‐analysis of clinical trials investigating the effects of (A) duration (week) of weight loss interventions on HDL-C. *WMD*: weighted mean difference, *HDL-C*: High-density lipoprotein cholesterol |

| 1. Duration    |
| --- |
| **Figure S18.** Forest plots from the meta‐analysis of clinical trials investigating the effects of (A) duration (week) of weight loss interventions on LDL-C. *WMD*: weighted mean difference, *LDL-C*: Low-density lipoprotein cholesterol |

| A) Duration   | B) Type   |
| --- | --- |
| **Figure S19.** Forest plots from the meta‐analysis of clinical trials investigating the effects of (A) duration (week) and (B) type of weight loss interventions on TC. *WMD*: weighted mean difference, *TC*: Total cholesterol | |

| A) Duration   | B) Type   |
| --- | --- |
| **Figure S20.** Forest plots from the meta‐analysis of clinical trials investigating the effects of (A) duration (week) and (B) type of weight loss interventions on ALT. *WMD*: weighted mean difference, *ALT*: Alanine transaminase | |

| A) Duration   | B) Type   |
| --- | --- |
| **Figure S21.** Forest plots from the meta‐analysis of clinical trials investigating the effects of (A) duration (week) and (B) type of weight loss interventions on AST. *WMD*: weighted mean difference, *AST*: Aspartate transaminase | |
